# H_2_-CO_2_ polymer electrolyte fuel cell that generates power while evolving CH_4_ at the Pt_0.8_Ru_0.2_/C cathode

**DOI:** 10.1038/s41598-021-87841-4

**Published:** 2021-04-16

**Authors:** Shofu Matsuda, Yuuki Niitsuma, Yuta Yoshida, Minoru Umeda

**Affiliations:** Department of Materials Science and Technology, Graduate School of Engineering, Nagaoka University of Technology, 1603-1 Kamitomioka, Nagaoka, Niigata 940-2188 Japan

**Keywords:** Carbon capture and storage, Fuel cells

## Abstract

Generating electric power using CO_2_ as a reactant is challenging because the electroreduction of CO_2_ usually requires a large overpotential. Herein, we report the design and development of a polymer electrolyte fuel cell driven by feeding H_2_ and CO_2_ to the anode (Pt/C) and cathode (Pt_0.8_Ru_0.2_/C), respectively, based on their theoretical electrode potentials. Pt–Ru/C is a promising electrocatalysts for CO_2_ reduction at a low overpotential; consequently, CH_4_ is continuously produced through CO_2_ reduction with an enhanced faradaic efficiency (18.2%) and without an overpotential (at 0.20 V vs. RHE) was achieved when dilute CO_2_ is fed at a cell temperature of 40 °C. Significantly, the cell generated electric power (0.14 mW cm^−2^) while simultaneously yielding CH_4_ at 86.3 μmol g^−1^ h^−1^. These results show that a H_2_-CO_2_ fuel cell is a promising technology for promoting the carbon capture and utilization (CCU) strategy.

## Introduction

Recently, carbon capture and utilization (CCU) methods have received significant levels of attention as technologies for effectively removing and utilizing atmospheric CO_2_^1,2^. These technologies are attractive approaches as they treat CO_2_ as an unused resource and convert it into value-added chemicals and fuels. Among them, electroreduction is a promising technique, and C_1_, C_2_, and C_2+_ products have reportedly been obtained using various electrocatalysts through different CO_2_ reduction mechanisms^3–13^. In particular, CO production at Au and Ag, and hydrocarbon production at Cu, have been successfully elucidated to follow multistep proton-coupled electron-transfer pathways^14,15^. These reactions exhibit relatively high faradaic efficiencies; however, they require large overpotentials, which is disadvantageous. Consequently, their energy-conversion efficiencies are low, despite their high faradaic efficiencies.

Developing methods that ensure that the CO_2_-electroreduction reaction occurs with a small overpotential and a high energy-conversion efficiency is important. In this regard, platinum group metals have the potential to realize overpotential-free CO_2_ reductions. There are many reports in which CO is adsorbed on a metal (CO_ads_) at a positive potential rather than its theoretical potential^16,17^. However, the further reduction of CO_ads_ is difficult because CO is strongly adsorbed to the metal through a donation–back-donation mechanism (Blyholder mechanism)^18^. To the best of our knowledge, the main product is H_2_ when a Pt electrocatalyst is used, even when it is negatively polarized^19–21^. We previously obtained the C1 compound by reducing CO_2_ near the theoretical potential in a proton-exchange-type membrane electrode assembly (MEA) with a carbon-supported platinum (Pt/C) electrocatalyst^22^. The use of a proton-exchange membrane and an ionomer was suggested to facilitate CO_2_ reduction; however, the C1 yield was quite low. We recently demonstrated that CH_4_ can be produced by the reduction of CO_2_ in the absence of an overpotential and with a faradaic efficiency of 6.8% using the MEA^23^. This CH_4_-generation reaction proceeds by a Langmuir–Hinshelwood (L–H) mechanism associated with CO_ads_ and H adsorbed on the metal (H_ads_):

1 $${\text{CO}}_{{{\text{ads}}}} + {\text{ 6 H}}_{{{\text{ads}}}} \to {\text{CH}}_{{4}} + {\text{ H}}_{{2}} {\text{O }} + { 7}^*,$$

where * represents an active site on the metal catalyst. It follows that placing CO_ads_ and H_ads_ in the appropriate ratio on the metal surface is important for CH_4_ production, and this is realized by controlling the CO_2_-feed concentration as well as the electrode potential in the case of a Pt catalyst^24^. Based on these techniques, we reported CO_2_ reduction at a Pt catalyst to generate CH_4_ with a faradaic efficiency of 12.3% at 0.16 V vs. RHE using 4 vol% CO_2_ diluted with Ar (Ref.^25^).

Here, the most noteworthy point is that the CH_4_-synthesis potential is almost the same as the theoretical potential, which is more positive than that for the hydrogen oxidation reaction (HOR). Therefore, power can be generated by an H_2_-CO_2_ fuel cell^26^ by combining the HOR and the CO_2_ reduction reaction (to generate CH_4_) as the anodic and cathodic reactions, respectively^25^. The H_2_-CO_2_ fuel cell is a promising CCU technology that utilizes CO_2_ as a resource to generate electricity while producing a valuable compound (CH_4_). However, the CH_4_ yield as well as the amount of power generated need to be increased to further develop this technology, and the efficient use of CO_ads_, which requires weakening the CO-metal bond, would represent a potential breakthrough toward this goal.

Pt-Ru alloy catalysts are known to impact the ligand effect, in which Ru affects the electronic state of CO_ads_ and weakens the CO-metal bond^27–30^. In our previous study, we investigated the reduction of CO_2_ using MEAs incorporated with Pt-Ru electrocatalysts and revealed that the CH_4_-generation efficiencies at the theoretical potential follow the order: Pt/C < Pt_0.5_Ru_0.5_/C < Pt_0.8_Ru_0.2_/C when 100 vol% CO_2_ was supplied^31^. For this reason, we expected that CH_4_ would be more-efficiently produced by the reduction of CO_2_ without an overpotential by combining both techniques, namely diluting the CO_2_ concentration and using a MEA with the Pt_0.8_Ru_0.2_/C electrocatalyst. In this work, we designed a polymer electrolyte fuel cell that incorporated a MEA with a Pt_0.8_Ru_0.2_/C cathode and a Pt/C anode. This paper reports the reduction of CO_2_ for the simultaneous production of CH_4_ and power by supplying CO_2_ and H_2_ to the cathode and anode, respectively.

## Results

### Effect of CO_2_ concentration on CH_4_ yield

**Figure 1:**
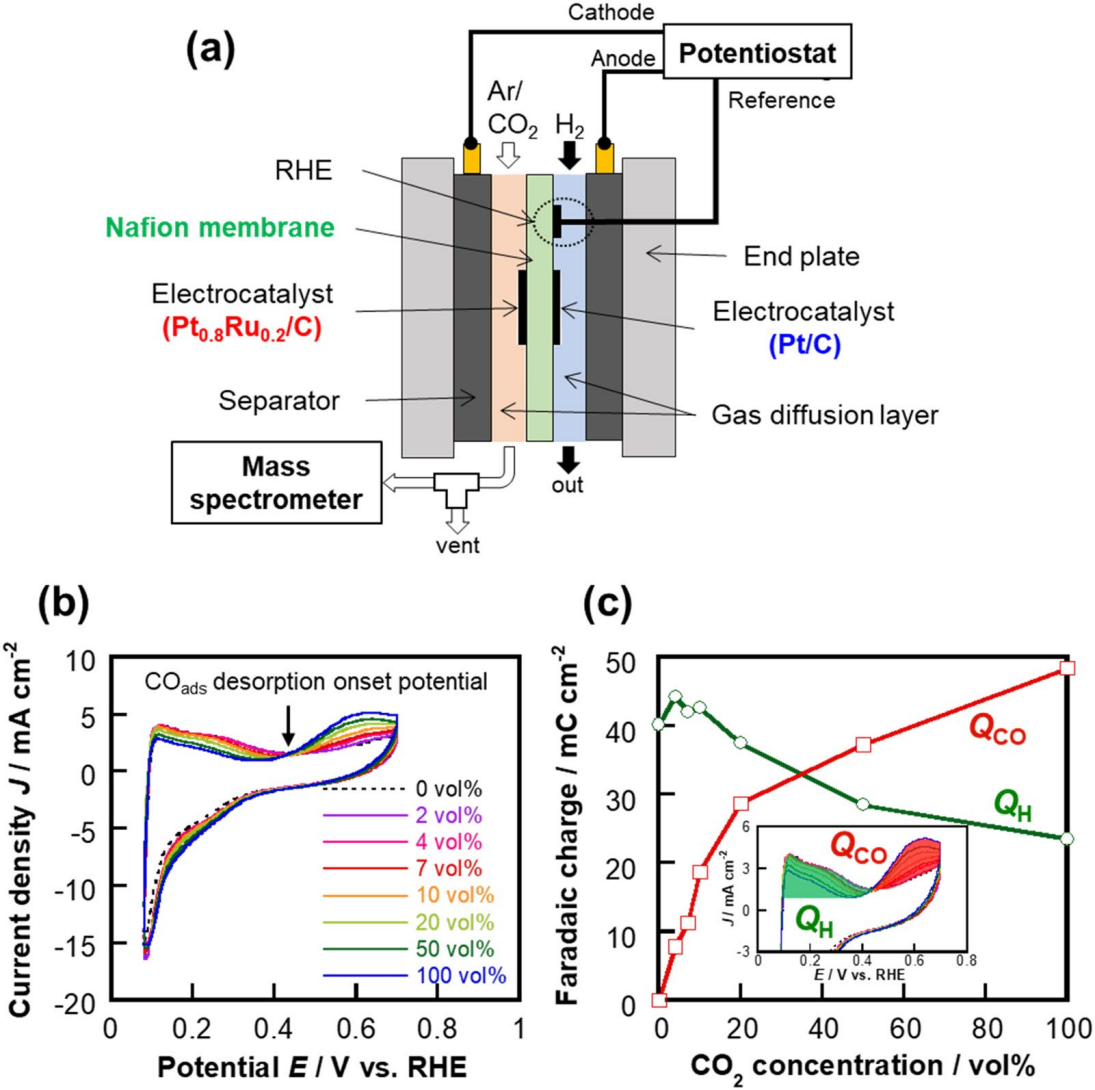
Schematic diagram and performance data. (**a**) Schematic diagram of the experimental setup used in this study. (**b**) Cathodic cyclic voltammograms at various concentrations of CO_2_ in Ar. (**c**) CO_2_-concentration-dependences of the faradaic charges of the oxidation-current peaks between 0.08 and 0.43 V (*Q*_H_) and between 0.43 and 0.70 V (*Q*_CO_) (vs. RHE) in the voltammograms shown in (**b**). The method used to calculate *Q*_H_ and *Q*_CO_ is shown in the inset in panel (**c**).

We first performed cyclic voltammetry (CV) to assess the cathodic reaction of the prepared cell (Fig. 1a) under various CO_2_ concentrations in Ar at a cell temperature of 40 °C. As shown in Fig. 1b, the oxidation current decreases with increasing CO_2_ concentration in the 0.08–0.43 V (vs. RHE) potential range, whereas it increases between 0.43 and 0.70 V (vs. RHE). Considering that the former and the latter are the oxidation currents that originate from H desorption and CO desorption, respectively^32^, this result reveals that the amounts of CO_ads_ and H_ads_ increase and decrease, respectively, with increasing CO_2_ concentration. The faradaic charges for H_ads_ (*Q*_H_) and CO_ads_ (*Q*_CO_) calculated from Fig. 1b are shown in Fig. 1c. In detail, *Q*_H_ and *Q*_CO_ were calculated as the integrated faradaic oxidation currents at 0.08–0.43 V (vs. RHE) and 0.43–0.70 V (vs. RHE), respectively, as depicted as the green filled area (for *Q*_H_) and the red filled area (for *Q*_CO_) in the inset in Fig. 1c. Based on Fig. 1c, a trade-off relationship between the *Q*_CO_ and *Q*_H_ are clearly observed. Therefore, the amounts of CO_ads_ and H_ads_ on the Pt_0.8_Ru_0.2_/C catalyst surface can be controlled by changing the concentration of CO_2_ supplied to the cathode. The onset potential for CO_ads_ desorption was determined, as shown by the black arrow in Fig. 1b, which provided a value of 0.43 V (vs. RHE) for Pt_0.8_Ru_0.2_/C, which is more negative than that for Pt/C (0.45 V vs. RHE^25^), suggesting that the Pt_0.8_Ru_0.2_/C electrocatalyst exhibits a lower CO-adsorption energy.

**Figure 2:**
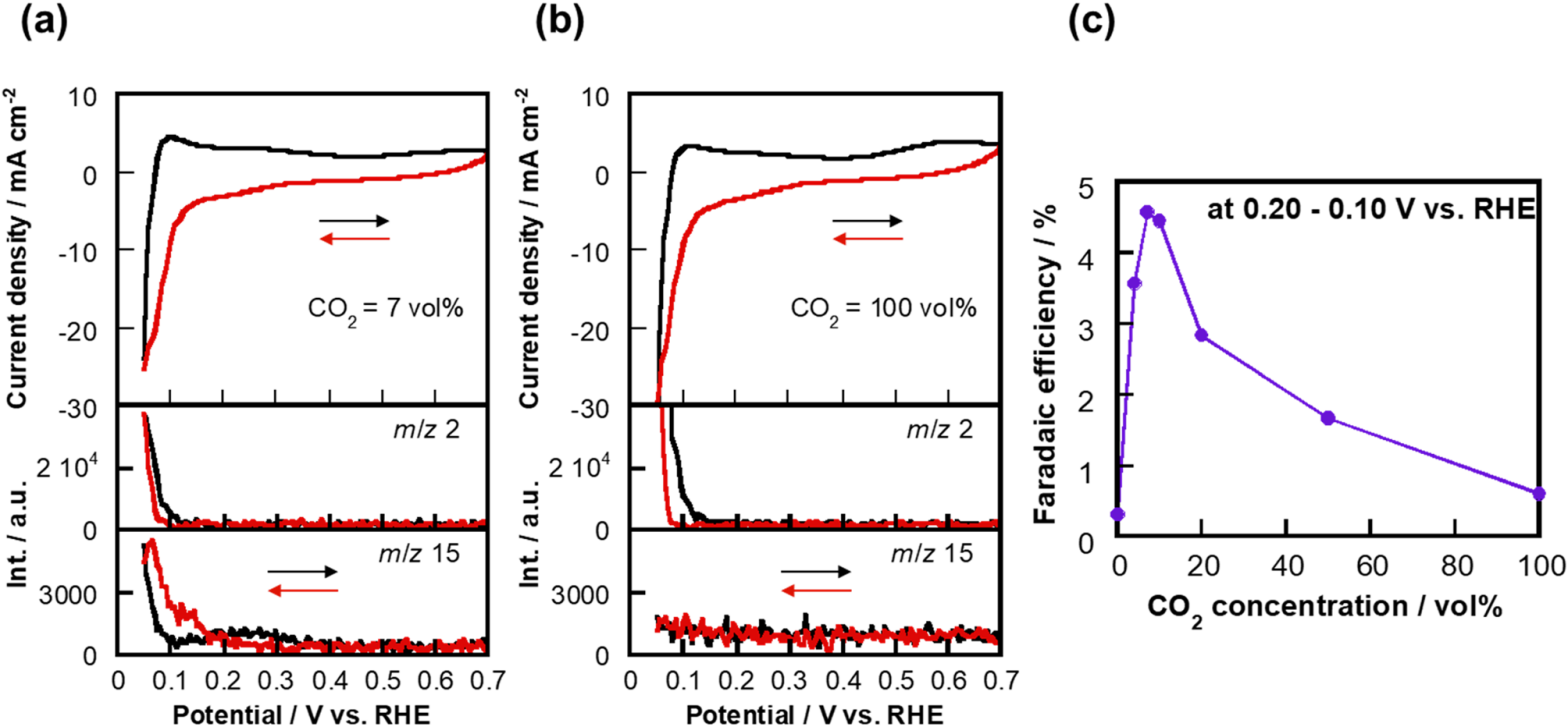
Cyclic voltammetry coupled with mass spectrometry. In-line *m*/*z* 2 (for H_2_) and 15 (for CH_4_) MS signals during CV at CO_2_ concentrations of: (**a**) 7 vol% and (**b**) 100 vol%. (**c**) Faradaic efficiency for the generation of CH_4_ as a function of CO_2_ concentration calculated by integrating the *m*/*z* 15-signals in the 0.20–0.10 V (vs. RHE) potential range during negative-scan CV.

CH_4_ generation from CO_2_ reduction at the Pt_0.8_Ru_0.2_/C electrocatalyst was next investigated. Figure 2a,b show cyclic voltammograms with in-line MS signals (*m*/*z* 2 and 15) at CO_2_ concentrations of 7 vol% and 100 vol%, respectively. The signal at *m*/*z* 15 was simultaneously detected with a reduction current at 7 vol% CO_2_ when the potential was below ~ 0.25 V (vs. RHE), whereas the *m*/*z* 15 signal only was weakly detected at 100 vol% CO_2_. The pattern in the mass spectrum of the cathodic output gas from the cell at 0.10 V (vs. RHE) in the 7 vol% CO_2_ atmosphere (Supplementary Fig. S1) is concordant with that of the CH_4_ standard gas; hence, the detected signal at *m*/*z* 15, which corresponds to CH_3_^+^ (not affected by H_2_O and CO_2_), is entirely derived from CH_4_ produced through the reduction of CO_2_. It should be noted that the signal at *m*/*z* 2 as hydrogen generation started to be detected at ~ 0.08 V (vs. RHE) at all CO_2_ concentrations. Figure 2c shows the dependence of the faradaic efficiency determined during CH_4_ generation on the CO_2_ concentration acquired during negative-potential-sweep CV between 0.20 and 0.10 V (vs. RHE). The faradaic efficiency was determined as a percentage of the methanogenic faradaic charge relative to the total faradaic charge. As a result, the highest faradaic efficiency of ~ 4.5% was calculated at 7 vol% CO_2_, which exceeds the efficiency for Pt/C (3.0% in a 5 vol% CO_2_ atmosphere^24^). On the other hand, the faradaic efficiency was only 0.61% in 100 vol% CO_2_, which corresponds to our previously reported efficiency^31^. Overall, we determined 7 vol% to be the preferred CO_2_ concentration for generating CH_4_ at the Pt_0.8_Ru_0.2_/C electrocatalyst.

### CH_4_-generation dependence on the CO_2_ electroreduction potential

**Figure 3:**
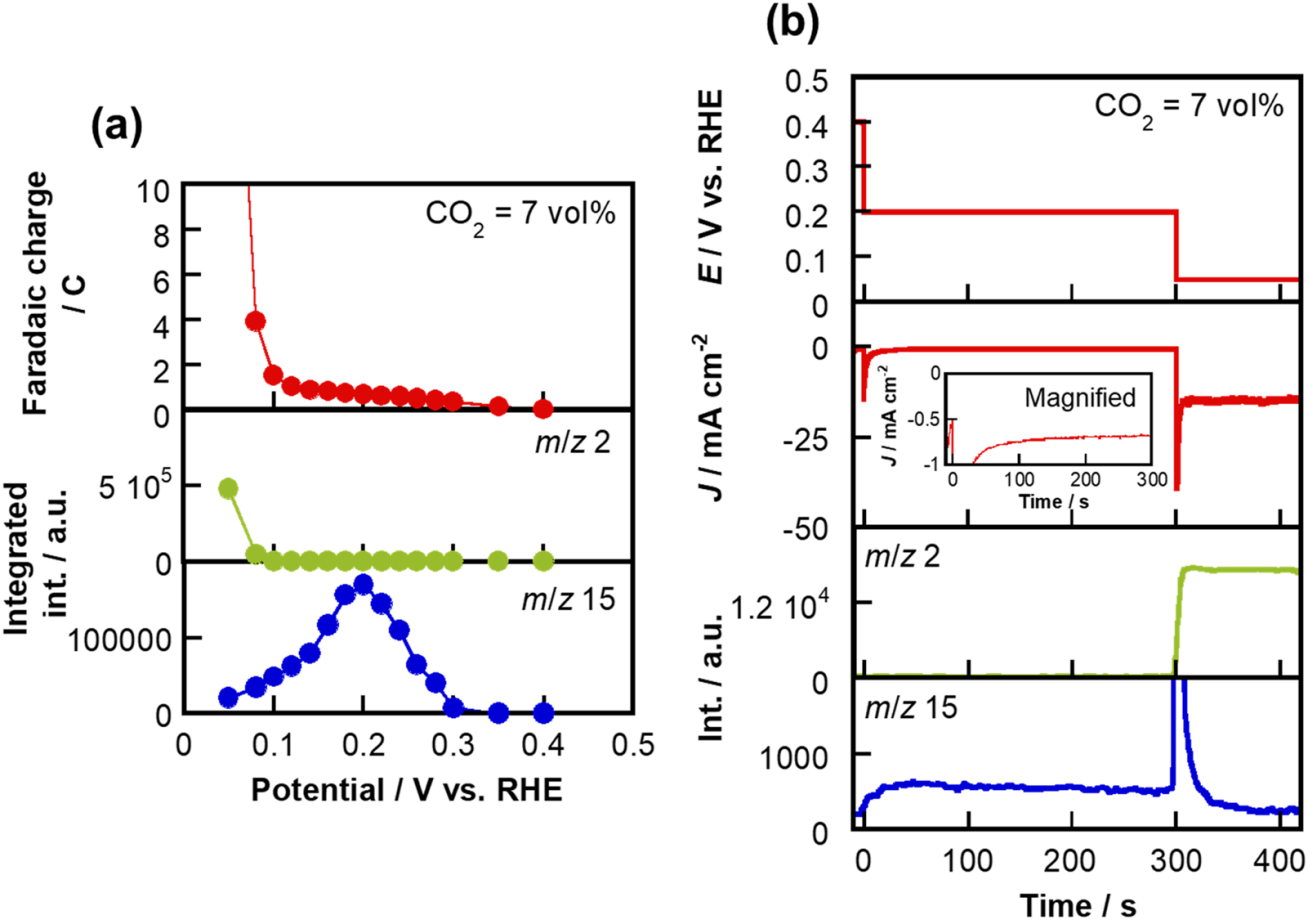
Stationary-potential CO_2_ reduction. (**a**) Potential dependence of faradaic charge, and integrated *m*/*z* 2 and 15 MS signals when held at each potential for 2 min at a CO_2_ concentration of 7 vol%. (**b**) Potential program applied to the cathode (upper), current response (middle), and in-line *m*/*z* 2 and 15 MS signals (lower) at 7 vol% CO_2_.

We next explored the optimum potential for CH_4_ production at the Pt_0.8_Ru_0.2_/C electrocatalyst with the supplied CO_2_ concentration fixed at 7 vol% under a potentio-static condition. The cathode potential was stepped 14 times in the negative direction in the 0.40–0.05 V (vs. RHE) range every 2 min. As shown in Fig. 3a, which was produced by analyzing Supplementary Fig. S2, the signal at *m*/*z* 15 for CH_4_ began to be detected at 0.30 V (vs. RHE), was most intense at around 0.20 V (vs. RHE), and began to decrease in intensity below this value. The cathode potential at which the maximum *m*/*z* 15 signal was observed was different under the potentio-dynamic (Fig. 2a) and potentio-static (Fig. 3a) conditions, probably be due to the slow reaction rate of CO_2_ → CH_4_. Meanwhile, the signal at *m*/*z* 2 for H_2_ was observed only at 0.08 V and 0.05 V (vs. RHE), where CH_4_ generation was suppressed. These results reveal that CH_4_ production occurs at a more positive potential than H_2_ evolution, with a maximum CH_4_ yield observed at 0.20 V (vs. RHE). The in-line MS signals (*m*/*z* 2 and 15) recorded during cyclic voltammetry at 0 vol% CO_2_ (100 vol% Ar) shown in Supplementary Fig. S3 reveal that the *m*/*z* 15 signal was hardly detected at all cathode potentials. Based on the standard CO_2_|CH_4_ electrode potential (0.169 V vs. SHE^33^), we successfully generated CH_4_ from CO_2_ in the absence of an overpotential in this study. Therefore, from the viewpoints of CH_4_ yield and product selectivity, 0.20 V vs. RHE was determined to be the preferred potential.

We subsequently directly stepped the potential from 0.40 to 0.20 V (vs. RHE). As shown in Fig. 3b, the intensity of the *m*/*z* 15 signal was observed to be almost constant when the potential was held at 0.20 V (vs. RHE). The faradaic efficiency of CH_4_ generation from CO_2_ reduction (CO_2_ + 8H^+^  + 8e^−^ → CH_4_ + 2H_2_O) between 240 and 299 s in Fig. 3b was calculated by the following formula:

2 $${\text{Faradaic efficiency }}\left( {\text{\% }} \right) = \frac{nmF}{{It}} \times 100,$$

where *n* is the number of reaction electrons, *m* is the molar number of CH_4_, *F* is the Faraday constant, *I* is the mean current, and *t* is the time. Significantly, the faradaic efficiency was 18.2%, which exceeds the previously reported Pt/C efficiency (12.3%^25^). Supplementary Fig. S4 shows gas chromatograms of the cathodic output gas from the cell in which the cathode potential was held at 0.20 V (vs. RHE). Based on the results obtained using flame-ionization and thermal-conductivity detectors, only CH_4_ was produced during CO_2_ reduction at Pt_0.8_Ru_0.2_/C, and the faradaic efficiency was calculated to be 17.6%, as detailed in Supplementary Information S4. Overall, continuous CH_4_ generation with enhanced efficiency and zero overpotential was achieved at a CO_2_ concentration of 7 vol% and a holding potential of 0.20 V (vs. RHE).

### Power generation as an H_2_-CO_2_ fuel cell

**Figure 4:**
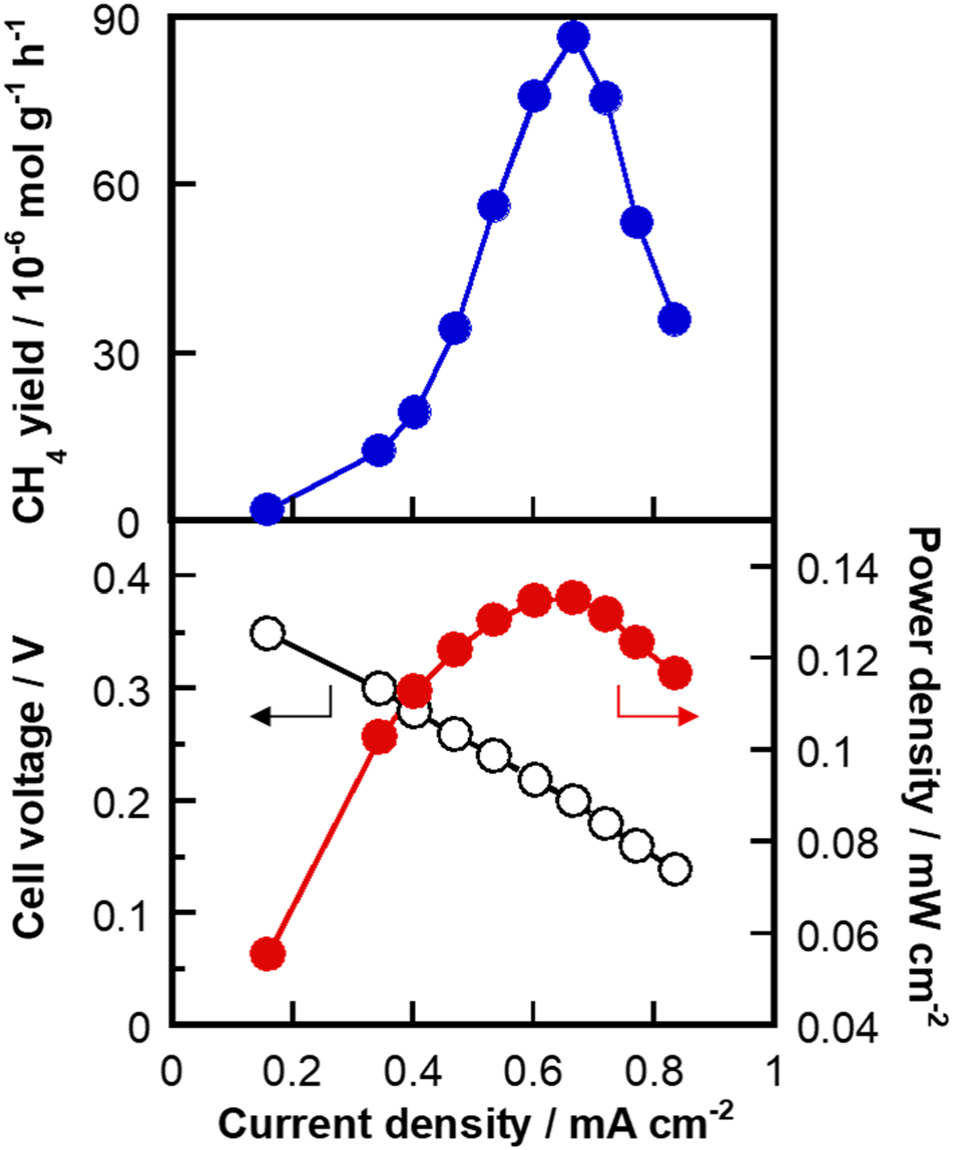
Cell performance. CH_4_ yield, cell voltage, and power density as functions of current density at a CO_2_ concentration of 7 vol%.

Figure 4 shows power generation characteristics as well as CH_4_-production rates determined from the data in Fig. 3a. The current densities shown in Fig. 4 are mean current densities (shown in Supplementary Fig. S2) for 60 s just before the next potential step. Figure 4 reveals that the power density (as an H_2_-CO_2_ fuel cell) and the CH_4_ yield rate exhibit similar trends, with maximum values of ~ 0.14 mW cm^−2^ and 86.3 μmol g^−1^ h^−1^, respectively, at a cell voltage of 0.20 V. Compared to a report on the formation of CH_3_OH (but not CH_4_) through CO_2_ reduction at Pt–Ru/C using a MEA^34^, the rate of CH_4_ production in this study is higher.

Finally, we compared the results obtained for Pt_0.8_Ru_0.2_/C with those for Pt/C. As listed in Table 1, the power density, CH_4_ yield rate, turnover frequency, and faradaic efficiency were ~ 10-, ~ 3.5-, ~ 5-, and ~ 1.5-times higher, respectively, when the Pt_0.8_Ru_0.2_/C electrocatalyst was used instead of the Pt/C electrocatalyst. Therefore, we demonstrated a H_2_-CO_2_ fuel cell that generates electric power while efficiently reducing CO_2_ to CH_4_.

**Table 1:** Comparing Pt_0.8_Ru_0.2_/C and Pt/C cathode electrocatalyst data for the H_2_-CO_2_ fuel cell.

	Pt_0.8_Ru_0.2_/C	Pt/C
Cell voltage/V	0.20	0.16
Power density/mW cm^−2^	0.14	0.015
CH_4_ yield rate/μmol g^−1^ h^−1^	86.3	26.4
Turnover frequency/nmol (ECSA)^−1^ s^−1^	0.73	0.15
Faradaic efficiency/%	18.2	12.3

Pt/C data are taken from our previous report^25^.

## Discussion

In the present study, we designed and demonstrated an H_2_-CO_2_ polymer-electrolyte fuel cell that generates CH_4_ from CO_2_ with enhanced efficiency, which was achieved by the strategic use of a Pt_0.8_Ru_0.2_/C cathodic catalyst. As mentioned in the introduction, the generation of CH_4_ through the reduction of CO_2_ follows the L–H mechanism involving CO_ads_ and H_ads_ (Ref.^23^). According to Eq. (1), this reaction theoretically proceeds at a CO_ads_-to-H_ads_ molar ratio of 1:6; however, this reaction proceeded when the ratio was 1:11 or higher for a Pt/C electrocatalyst^24^, with the best ratio reported to be 1:18 (Ref.^24^). On the other hand, this ratio at the Pt_0.8_Ru_0.2_/C electrocatalyst was determined to be 1:8 at a CO_2_ concentration of 7 vol% according to the following equation:

3 $${\text{CO}}_{{{\text{ads}}}} :{\text{H}}_{{{\text{ads}}}} = \left( {\frac{{Q_{CO} }}{2}} \right):Q_{H} .$$

Hence, CO_ads_ can be used to efficiently generate CH_4_ at Pt_0.8_Ru_0.2_/C because the ratio is close to the theoretical value of 1:6, which leads to a higher faradaic CH_4_-production efficiency, and was achieved by the lower CO-adsorption energy associated with the Pt_0.8_Ru_0.2_/C electrocatalyst.

The sustained generation of CH_4_ at 0.20 V (vs. RHE) (Fig. 3b) is also related to the CO_ads_-to-H_ads_ ratio. Based on Supplementary Fig. S5, this ratio was determined to be 1:7 after holding at a potential of 0.20 V (vs. RHE) for 5 min at 7 vol% CO_2_. Therefore, CH_4_ is continuously generated at Pt_0.8_Ru_0.2_/C because the CO_ads_-to-H_ads_ ratio was slightly different when the potential was held at 0.20 V (vs. RHE). It should be noted that the CH_4_-production reaction proceeds at a more-positive potential than its theoretical potential. Although the reason is unclear at present, there is one possible explanation for this observation. In our system, the CO_2_|CH_4_ equilibrium potential is influenced by the proton activity of the Nafion membrane electrolyte. As shown in Supplementary Fig. S3, the H_2_-evolution onset potential is ~ 0.08 V (vs. RHE); this value does not correspond to the theoretical potential (0.00 V vs. SHE^33^). In addition, the proton activity of the Nafion membrane has been reported to be different to that observed under SHE conditions^35–37^. Importantly, the CH_4_-generation reaction is likely to occur through a sequential CO_2_ → CO_ads_ → CH_4_ reduction process, rather than through a one-step process (CO_2_ → CH_4_). Supplementary Fig. S6 shows that the onset potential for the formation of CO_ads_ from CO_2_ through reduction at Pt_0.8_Ru_0.2_/C in 7 vol% CO_2_ is 0.375 V (vs. RHE). Hence, an electrode potential that progresses the CO_ads_ → CH_4_ process can drive the overall reaction.

H_2_-CO_2_ fuel cells function when platinum group metals are employed as cathodic catalysts. In other words, a H_2_-CO_2_ fuel cell does not function using “active” electrocatalysts composed of only Cu, Au, and Ag, as well as their alloys, because CO_2_ reduction proceeds at a more negative potential than that for HOR (the overpotential is large)^38^. It should be noted that the highest faradaic CH_4_-yield efficiency was only 18.2% in this study, which is insufficient for practical applications. The reason for this low efficiency has not yet been clarified; one possible process that contributes to the rest efficiency involves the formation of CO_ads_ and H_ads_, which are not associated with CH_4_ generation. Hence, further increasing the efficiency through catalyst design, including optimizing the Ru ratio in the Pt-Ru catalyst, will be important. One significant advantage of this technology is that the CO_2_-conversion reaction that produces CH_4_ occurs at a lower temperature (40 °C) than that used in chemical methanation technology^39^ and CO_2_-utilization technologies that rely on solid oxide electrolytes^40^ and molten solts^41^, which require temperature of several hundreds of degrees.

In conclusion, this work provides a novel approach to H_2_-CO_2_ fuel cells as a CO_2_-utilization technology. CH_4_ is produced continuously by the reduction of CO_2_ in the absence of an overpotential (at 0.20 V vs. RHE) in a cell that uses a MEA with a Pt_0.8_Ru_0.2_/C cathode at a CO_2_ concentration of 7 vol% and a cell temperature of 40 °C, and electrical energy is generated by combining the CO_2_-reduction and H_2_-oxidation (at a Pt/C anode) reactions. These results facilitate the carbon utilization strategy, although further investigations are necessary before it can be considered for practical applications.

## Methods

### Materials

Pt_0.8_Ru_0.2_/C (42.5 wt%; TECRuE43) and Pt/C (46.2 wt%; TEC10E50E) electrocatalyst powders were obtained from Tanaka Kikinzoku Kogyo Co., Ltd. Nafion-117 membranes (0.18-mm thick) were purchased from DuPont and boiled successively in Milli-Q water, 0.5 M H_2_O_2_, 0.5 M H_2_SO_4_, and Milli-Q water (1 h each) prior to use. All chemicals (H_2_O_2_, H_2_SO_4_, acetone, 2-propanol, methanol, and 5 wt% Nafion solution) were obtained from the Fujifilm Wako Pure Chemical Corporation. Water-repellent carbon paper (TGP-H-060H) was purchased from Toray Industries, Inc, and polymer electrolyte cell components (gasket, separator with parallel flow paths, and stainless steel plate) were purchased from Miclab.

### Cell fabrication

A polymer electrolyte fuel cell (PEFC) was fabricated by essentially following the same procedure reported previously^23–25^, with the exception that Pt_0.8_Ru_0.2_/C was used as the cathode instead of Pt/C. Briefly, a 6 × 6 cm^2^ Nafion 117 membrane and 3 × 3 cm^2^ pieces of carbon paper pretreated with acetone were used as the proton-exchange membrane and gas diffusion layers, respectively. The electrocatalyst dispersion was prepared by mixing the Pt_0.8_Ru_0.2_/C catalyst with 5 wt% Nafion (1:1 v/v) and an aqueous solution containing 1:2:1 (w/w/w) 2-propanol, methanol, and Milli-Q water, followed by spraying onto one piece carbon paper to prepare the cathode. The anode was prepared by spraying a Pt/C electrocatalyst dispersion onto another piece of carbon paper. The amount of loaded metal and the apparent electrode surface area were 1.0 mg cm^−2^ and 9.0 cm^2^, respectively, on both electrodes. The MEA was prepared by bringing these electrodes into contact with each side of the Nafion-117 membrane, followed by hot-pressing at 140 °C with a 4.5 kN load for 10 min. It should be noted that the Pt/C electrocatalyst dispersion was dropped onto the Nafion 117 membrane on the anode side to provide a reference reversible hydrogen electrode (RHE). Finally, the MEA, gasket, separator, and stainless steel plate were assembled to complete the PEFC used in this study, as shown in Fig. 1a.

### Electrochemical CO_2_ reduction and product analysis

A schematic diagram of the experimental setup used in this study is shown in Fig. 1a. Electrochemical experiments were conducted using a PEFC-operating apparatus (FCG-20S, ACE), a potentiostat/galvanostat (HA-310, Hokuto Denko), and a function generator (HB-104, Hokuto Denko). Fully humidified 100 vol% H_2_ and CO_2_ diluted with Ar (CO_2_ concentration: 0, 4, 7, 10, 20, 50, and 100 vol%) gas were fed to the anode and cathode at 50 cm^3^ min^−1^, respectively, in all experiments. Fully humidified 100 vol% H_2_ gas was supplied to the reference electrode at 10 cm^3^ min^−1^. The cell temperature was set to 40 °C because the cell humidification at least 40 °C is required to operate the MEA. The H_2_, CO_2_, and Ar gases were 99.999%, 99.995%, and 99.998% pure, respectively. Before electrochemical measurements, the cathodic potential sweep in the 0.05–0.70 V (vs. RHE) range at 50 mV s^−1^ was repeated until the current–potential curve of the cathode became stable. The initial cathode potential during introduction of CO_2_-containing gas was ~ 0.13 V (vs. RHE). The electrochemical surface area (ECSA) of the cathode electrocatalyst was obtained to be 0.294 m^2^ according to the hydrogen adsorption method^30,42,43^. The cathodic potential was scanned in the 0.08–0.70 V (vs. RHE) range at 10 mV s^−1^ during CV. It should be noted that that a 0.05–0.70 V (vs. RHE) potential range was used for CV with in-line product analysis. In the potential-step experiment, the cathodic potential was stepped through 14 levels in the 0.40–0.05 V (vs. RHE) range in the negative direction every 2 min at a CO_2_ concentration of 7 vol%. In addition, the cathodic potential was directly stepped from 0.40 to 0.20 V (vs. RHE) at 7 vol% CO_2_ and held there for 5 min, after which it was stepped to 0.05 V (vs. RHE). In-line mass spectrometry (MS) was carried out during the electrochemical experiments by introducing the cathode exhaust gas directly to a mass spectrometer (JMS-Q1050GC, JEOL). The ionization voltage was 23 eV. Note that the lag time for in-line MS product detection was adjusted by the H_2_ evolution response (7 s). A calibration curve, which was obtained using CH_4_ gas (purity: 99.999%) diluted with Ar, was used to calculate the amount of CH_4_ generated, for determining the faradaic efficiencies and CH_4_-yield rates. All the current densities were calculated using the apparent electrode surface area (9.0 cm^2^).

## Supplementary Information

Supplementary Information

## References

[1] Hepburn C (2019). The technological and economic prospects for CO_2_ utilization and removal. Nature.

[2] Abanades JC, Rubin ES, Mazzotti M, Herzog HJ (2017). On the climate change mitigation potential of CO_2_ conversion to fuels. Energy Environ. Sci..

[3] Liu M (2016). Enhanced electrocatalytic CO_2_ reduction via field-induced reagent concentration. Nature.

[4] Hossain, M. H., Wen, J. & Chen., A. Unique copper and reduced graphene oxide nanocomposite toward the efficient electrochemical reduction of carbon dioxide. *Sci. Rep.* **7**, 3184. 10.1038/s41598-017-03601-3 (2017). 10.1038/s41598-017-03601-3 PMC5466611 28600564

[5] Liu S (2017). Shape-dependent electrocatalytic reduction of CO_2_ to CO on triangular silver nanoplates. J. Am. Chem. Soc..

[6] Nguyen DLT (2017). Selective CO_2_ reduction on zinc electrocatalyst: The effect of zinc oxidation state induced by pretreatment environment. ACS Sustainable Chem. Eng..

[7] Li F, Chen L, Knowles PG, MacFarlane DR, Zhang J (2017). Hierarchical mesoporous SnO_2_ nanosheets on carbon cloth: a robust and flexible electrocatalyst for CO_2_ reduction with high efficiency and selectivity. Angew. Chem. Int. Ed..

[8] Wu Y, Jiang Z, Lu X, Liang Y, Wang H (2019). Domino electroreduction of CO_2_ to methanol on a molecular catalyst. Nature.

[9] Choi J (2019). Energy efficient electrochemical reduction of CO_2_ to CO using a three-dimensional porphyrin/graphene hydrogel. Energy Environ. Sci..

[10] Zhang E (2019). Bismuth single atoms resulting from transformation of metal−organic frameworks and their use as electrocatalysts for CO_2_ reduction. J. Am. Chem. Soc..

[11] Todoroki N, Tei H, Tsurumaki H, Miyakawa T, Inoue T, Wadayama T (2019). Surface atomic arrangement dependence of electrochemical CO_2_ reduction on gold: online electrochemical mass spectrometric study on low-index Au(*hkl*) Surfaces. ACS Catal..

[12] Kim T, Palmore TR (2020). A scalable method for preparing Cu electrocatalysts that convert CO_2_ into C_2+_ products. Nat. Commun..

[13] Arán-Ais RM (2020). Imaging electrochemically synthesized Cu_2_O cubes and their morphological evolution under conditions relevant to CO_2_ electroreduction. Nat. Commun..

[14] Kortlever R, Shen J, Schouten KJP, Calle-Vallejo F, Koper MTM (2015). Catalysts and reaction pathways for the electrochemical reduction of carbon dioxide. J. Phys. Chem. Lett..

[15] Peterson AA, Abild-Pedersen F, Studt F, Rossmeisl J, Nørskov JK (2010). How copper catalyzes the electroreduction of carbon dioxide into hydrocarbon fuels. Energy Environ. Sci..

[16] Giner J (1963). Electrochemical reduction of CO_2_ on platinum electrodes in acid solutions. Electrochim. Acta.

[17] Hori Y (2008). Electrochemical CO_2_ reduction on metal electrodes. Mod. Aspects Electrochem..

[18] Koper MTM, van Santen RA (1999). Electric field effects on CO and NO adsorption at the Pt(111) surface. J. Electroanal. Chem..

[19] Hori Y, Wakebe H, Tsukamoto T, Koga O (1994). Electrocatalytic process of CO selectivity in electrochemical reduction of CO_2_ at metal electrodes in aqueous media. Electrochim. Acta.

[20] Kuhl KP, Hatsukade T, Cave ER, Abram DN, Kibsgaard J, Jaramillo TF (2014). Electrocatalytic conversion of carbon dioxide to methane and methanol on transition metal surfaces. J. Am. Chem. Soc..

[21] Lee, J., Lim, J., Roh, C.-W., Whang, H. S. & Lee, H. Electrochemical CO_2_ reduction using alkaline membrane electrode assembly on various metal electrodes. *J. CO*_*2*_* Util.* **31**, 244–250. 10.1016/j.jcou.2019.03.022 (2019).

[22] Shironita S, Karasuda K, Sato M, Umeda M (2013). Feasibility investigation of methanol generation by CO_2_ reduction using Pt/C-based membrane electrode assembly for a reversible fuel cell. J. Power Sources.

[23] Umeda M, Niitsuma Y, Horikawa T, Matsuda S, Osawa M (2020). Electrochemical reduction of CO_2_ to methane on platinum catalysts without overpotentials: strategies for improving conversion efficiency. ACS Appl. Energy Mater..

[24] Matsuda S, Tamura S, Yamanaka S, Niitsuma Y, Sone Y, Umeda M (2020). Minimization of Pt-electrocatalyst deactivation in CO_2_ reduction using a polymer electrolyte cell. React. Chem. Eng..

[25] Umeda M, Yoshida Y, Matsuda S (2020). Highly selective methane generation by carbon dioxide electroreduction on carbon-supported platinum catalyst in polymer electrolyte fuel cell. Electrochim. Acta.

[26] Umeda M, Sato M, Maruta T, Shironita S (2013). Is power generation possible by feeding carbon dioxide as reducing agent to polymer electrolyte fuel cell?. J. Appl. Phys..

[27] Waszczuk P (2001). Adsorption of CO poison on fuel cell nanoparticle electrodes from methanol solutions: A radioactive labeling study. J. Electroanal. Chem..

[28] Christoffersen E, Liu P, Ruban A, Skriver HL, Nørskov JK (2001). Anode materials for low-temperature fuel cells: A density functional theory study. J. Catal..

[29] Wakisaka M, Mitsui S, Hirose Y, Kawashima K, Uchida H, Watanabe M (2006). Electronic structures of Pt-Co and Pt-Ru alloys for CO tolerant anode catalysts in polymer electrolyte fuel cells studied by EC-XPS. J. Phys. Chem. B.

[30] Furukawa H, Matsuda S, Tanaka S, Shironita S, Umeda M (2018). CO_2_ electroreduction characteristics of Pt-Ru/C powder and Pt-Ru sputtered electrodes under acidic condition. Appl. Surf. Sci..

[31] Niitsuma Y, Sato K, Matsuda S, Shironita S, Umeda M (2019). CO_2_ reduction performance of Pt-Ru/C electrocatalyst and its power generation in polymer electrolyte fuel cell. J. Electrochem. Soc..

[32] Shironita S, Karasuda K, Sato K, Umeda M (2013). Methanol generation by CO_2_ reduction at a Pt-Ru/C electrocatalyst using a membrane electrode assembly. J. Power Sources.

[33] Bard AJ, Parsons R, Jordan J (1985). Standard potentials in aqueous solution.

[34] Sebastián D (2017). CO_2_ reduction to alcohols in a polymer electrolyte membrane co-electrolysis cell operating at low potentials. Electrochim. Acta.

[35] Seger B, Vinodgopal K, Kamat PV (2007). Proton activity in Nafion films: Probing exchangeable protons with methylene blue. Langmuir.

[36] Umeda M, Sayama K, Maruta T, Inoue M (2013). Proton activity of Nafion 117 membrane measured from potential difference of hydrogen electrodes. Ionics.

[37] Brightman E, Pasquier D (2017). Measurement and adjustment of proton activity in solid polymer electrolytes. Electrochem. Commun..

[38] Zhang W (2018). Progress and perspective of electrocatalytic CO_2_ reduction for renewable carbonaceous fuels and chemicals. Adv. Sci..

[39] Wang W, Wang S, Ma X, Gong J (2011). Recent advances in catalytic hydrogenation of carbon dioxide. Chem. Soc. Rev..

[40] Lu, J. et al. Highly efficient electrochemical reforming of CH_4_/CO_2_ in a solid oxide electrolyser. *Sci. Adv.* **4**, eaar5100. 10.1126/sciadv.aar5100 (2018). 10.1126/sciadv.aar5100 PMC5903906 29670946

[41] Banerjee A, Dick GR, Yoshino T, Kanan MW (2016). Carbon dioxide utilization via carbonate-promoted C-H carboxylation. Nature.

[42] Trasatti S, Petrii OA (1992). Real surface area Measurements in electrochemistry. J. Electroanal. Chem..

[43] Wang H (2016). Role of Ru oxidation degree for catalytic activity in bimetallic Pt/Ru nanoparticles. J. Phys. Chem. C.

