# Supplementary material for: H2-CO2 polymer electrolyte fuel cell that generates power while evolving CH4 at the Pt0.8Ru0.2/C cathode
Source: Sci Rep. 2021 Apr 16;11:8382. doi: 10.1038/s41598-021-87841-4 (PMC8052373; doi:10.1038/s41598-021-87841-4)
Supplement: Supplementary file 1 — Supplementary Information [file 41598_2021_87841_MOESM1_ESM.pdf]

Supplementary Information for

H_2-CO_2 polymer electrolyte fuel cell that generates power while evolving CH_4 at the $Pt_{0.8}Ru_{0.2}$/C cathode

Shofu Matsuda, Yuuki Niitsuma, Yuta Yoshida, Minoru Umeda *

Department of Materials Science and Technology, Graduate School of Engineering, Nagaoka University of Technology, 1603-1 Kamitomioka, Nagaoka, Niigata 940-2188, Japan

* Corresponding author: mumeda@vos.nagaokaut.ac.jp (M. Umeda)

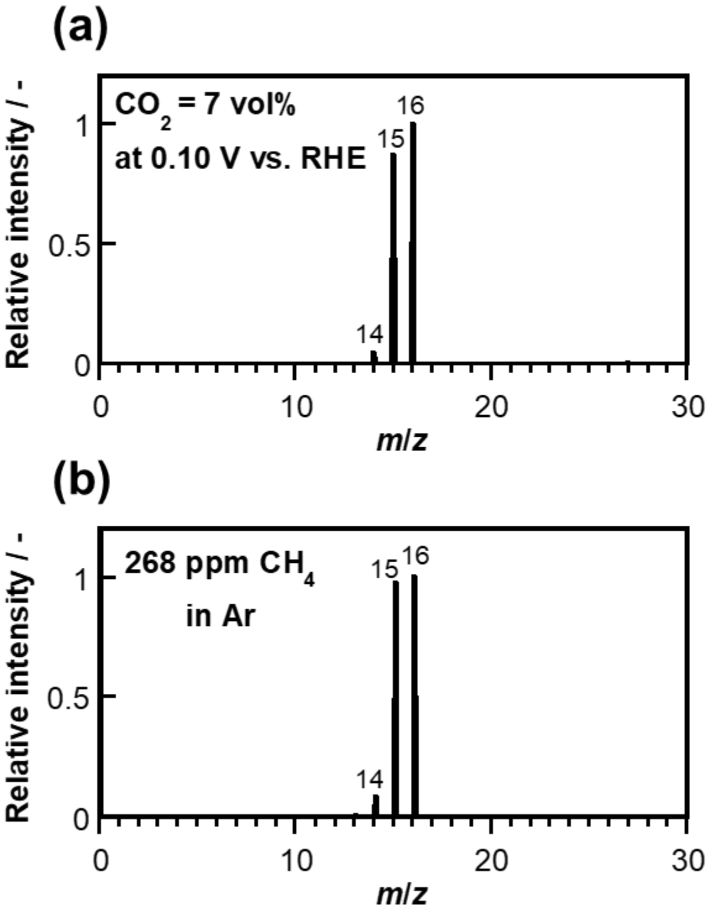

Fig. S1. Mass spectra. Mass spectra of (**a**) the cathodic output gas from the cell at 0.10 V (vs. RHE) during negative-scan CV in a 7 vol% CO_2 atmosphere (Fig. 2a) and (**b**) 268 ppm CH_4 standard gas diluted with Ar.

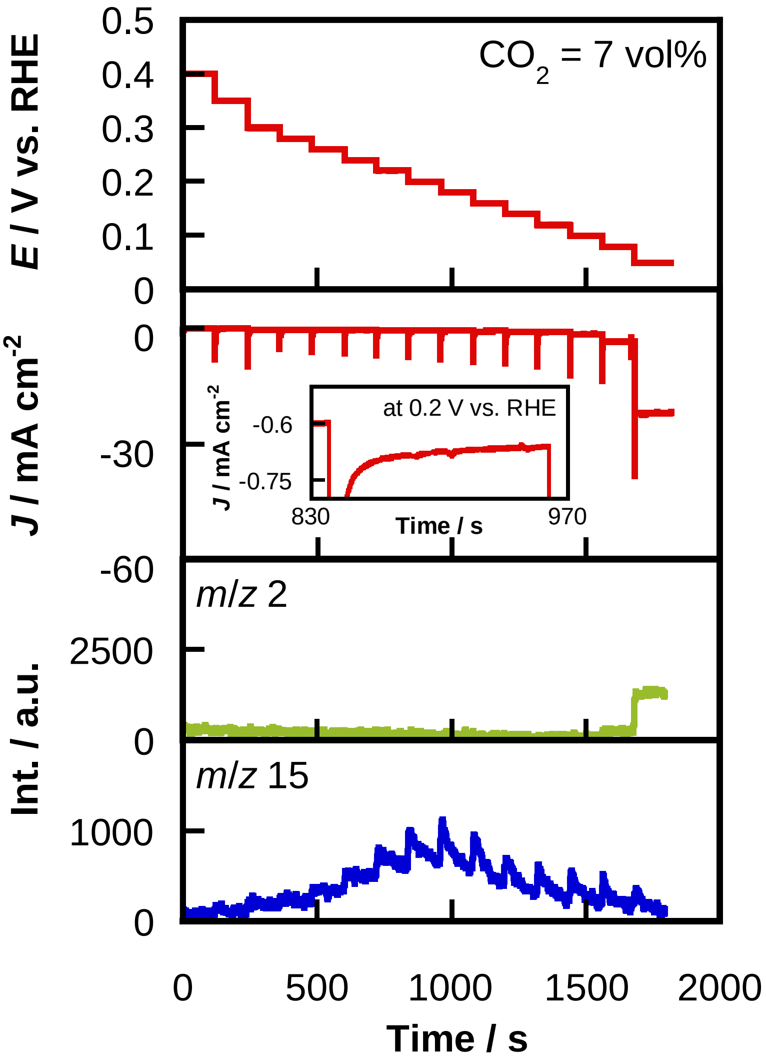

Fig. S2. Potential program and MS-signal responses. Potential program applied to the cathode (upper), current response (middle), and time-dependent in-line *m*/*z* 2 and 15 MS signal intensities (lower) in a 7 vol% CO_2 atmosphere.

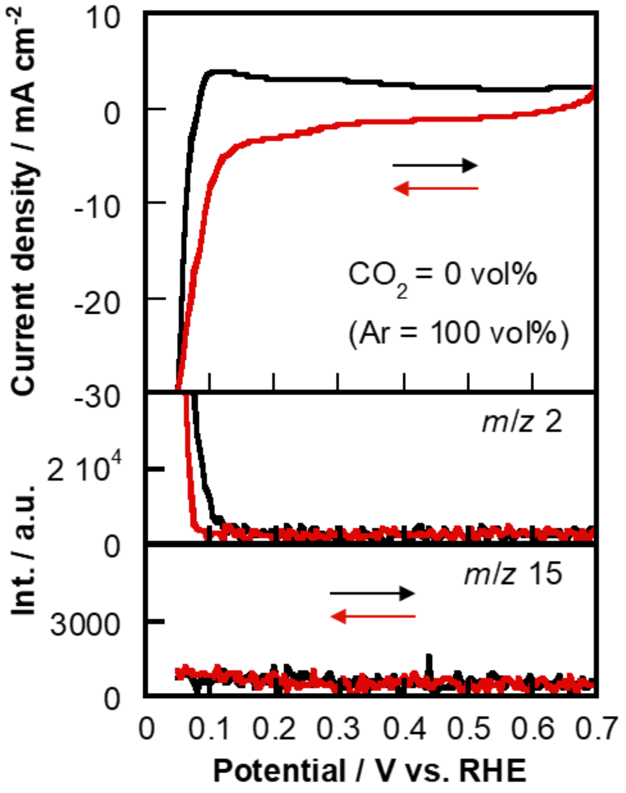

Fig. S3. Product analysis during CV. In-line *m*/*z* 2 (for H_2) and 15 (for CH_4) MS signals during cyclic voltammetry at a CO_2 concentration of 0 vol% (100 vol% Ar).

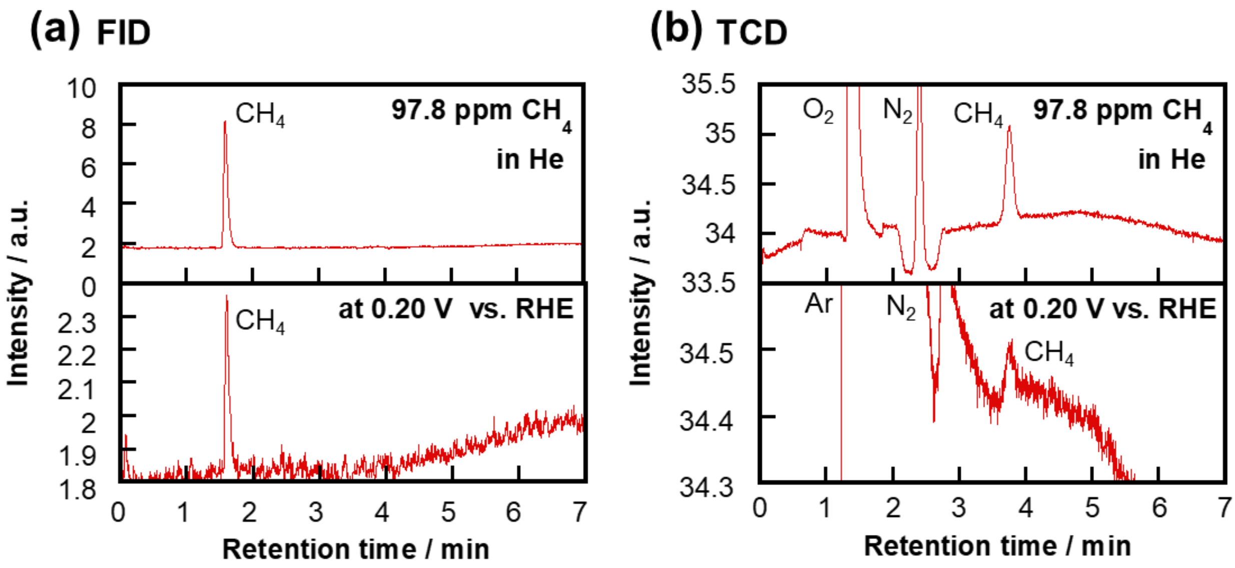

Fig. S4. Gas chromatography. Gas chromatograms of (upper) the 97.8 ppm CH_4 standard gas in He and (lower) the cathodic output gas from the cell during potential holding at 0.20 V (vs. RHE) in a 7 vol% CO_2 atmosphere using (**a**) a flame ionization detector (FID) and (**b**) a thermal conductivity detector (TCD). A 6890 series gas chromatograph (Agilent Technology Inc.) and a DB-WAX capillary column (Agilent Technology Inc.) were used in this study. Based on Fig. S4(a), the faradaic efficiency of CH_4 generation from CO_2 reduction was calculated using a following formula described in the literature (Luo, Jing-Li. *J. Am. Chem. Soc.* **139**, 2160–2163 (2017).):

$$\text{Faradaic efficiency (\%)} = \frac{0.4992 \times V\left(\frac{mL}{min}\right) \times v(vol\%)}{I(A)} \times 100,$$

where V is the gas flow rate, v is the volume concentration of CH_4, and I is the steady current. Since the steady current in this experiment was -1.2 mA, the faradaic efficiency was determined as 17.6%.

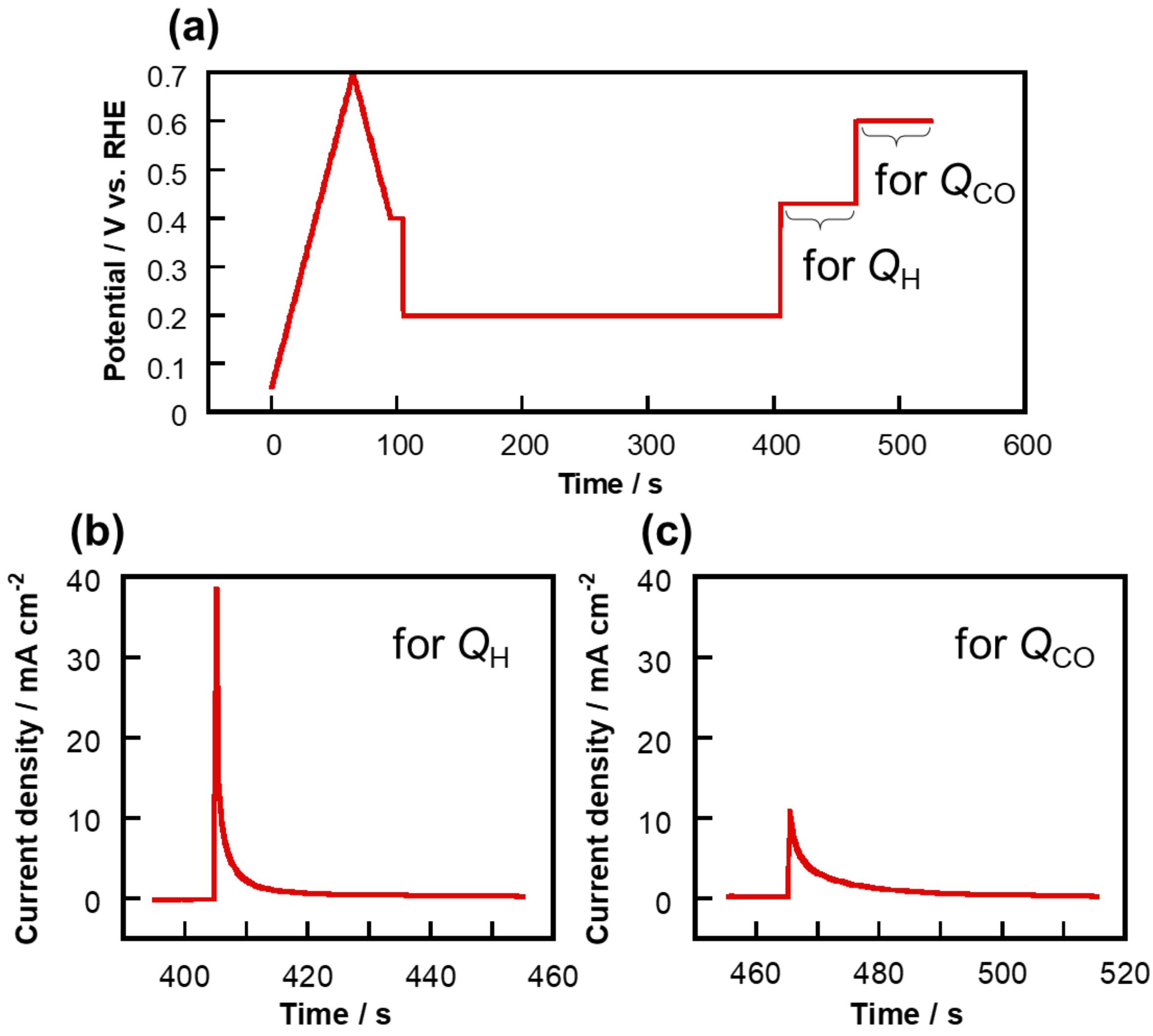

Fig. S5. Potential program and current responses. (**a**) Potential program applied in this experiment and the corresponding current responses used to determine (**b**) Q_H and (**c**) Q_{CO}. To determine why CH_4 was continuously generated during CO_2 reduction at 0.20 V (vs. RHE), we evaluated the CO_{ads}-to-H_{ads} ratio after holding for 5 min at 0.20 V (vs. RHE) in a 7 vol% CO_2 atmosphere, after which the potential was stepped to 0.43 V, which is the CO_{ads}-desorption onset potential, which enabled the faradaic charge associated with H desorption (Q_H) to be determined. After holding for 50 s hold at 0.43 V (vs. RHE) the potential was then stepped to 0.60 V (vs.

RHE) in order to determine the faradaic charge associated with CO desorption (Q_{CO}). Based on the values of Q_H and Q_{CO} determined from Figs. S5b and S5c, the CO_{ads}-to-H_{ads} ratio was calculated to be 1:7 using equation (3).

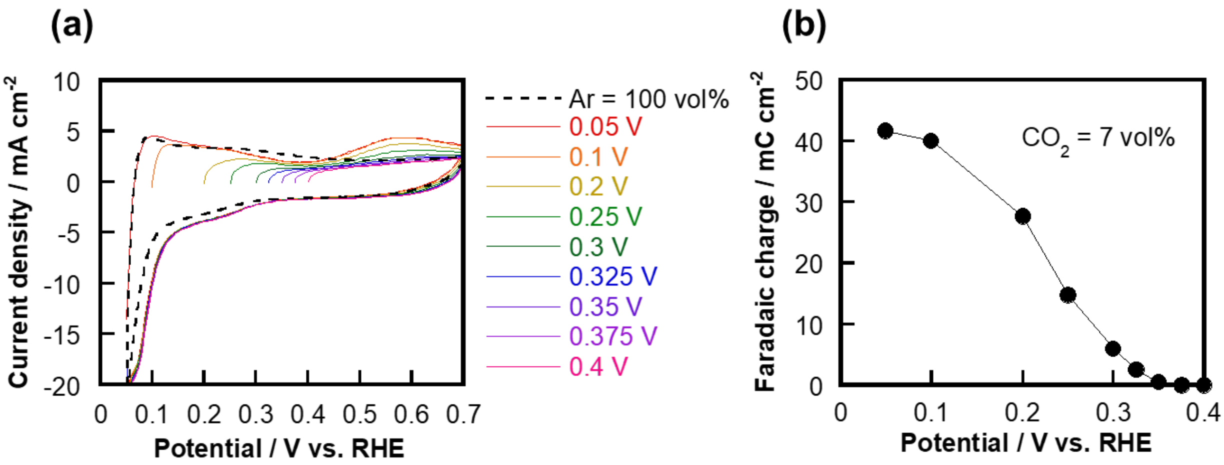

Fig. S6. Cyclic voltammograms and faradaic charges. (**a**) Cyclic voltammograms acquired after holding at each potential for 5 min and (**b**) dependence of faradaic charge (0.43–0.70 V vs. RHE in the cyclic voltammograms in (a)) on the hold potential at a CO_2 concentration of 7 vol% and a cell temperature of 40°C using the $Pt_{0.8}Ru_{0.2}$/C cathode catalyst. The cathodic potential in Fig. S6a was swept in the positive direction at 10 mV·s^{-1} after being held for 5 min at 0.05–0.40 V (vs. RHE) in 7 vol% CO_2. As a result, the CO desorption peak in the 0.43–0.70 V (vs. RHE) potential range decreased in size as the hold potential moved in the positive direction, and then disappeared at 0.375 V vs. RHE. The calculated faradaic charges derived from the oxidation current for CO desorption shown in Fig. S6a decreased in magnitude as the hold potential was positively shifted, and became 0.0 mC·cm^{-2} at 0.375 V vs. RHE, as shown in Fig. S6b. It should be noted that the onset potential for the formation of CO_{ads} through the reduction of CO_2 is defined as the hold potential which the faradaic charge derived from the CO-desorption oxidation

disappears. Therefore, the onset potential for the formation of CO_{ads} at the $Pt_{0.8}Ru_{0.2}/C$ electrocatalyst in 7 vol% CO_2 was determined to be 0.375 V (vs. RHE).
